# Effects of Nicotinamide Riboside Supplementation on Postmortem Mitochondrial Functionality and Apoptotic Activation

**DOI:** 10.3390/metabo15010031

**Published:** 2025-01-08

**Authors:** Chen Zhu, Luke A. Whitcomb, Adam J. Chicco, Morgan E. Gravely, Hanna M. Alcocer, Daniela A. Alambarrio, John M. Gonzalez, Colton L. Smith, Mahesh N. Nair, Huey Yi Loh, Terry E. Engle, Arya Niraula, Chaoyu Zhai

**Affiliations:** 1 Department of Animal Science, University of Connecticut, Storrs, CT 06269, USA; chen.3.zhu@uconn.edu (C.Z.); arya.niraula@uconn.edu (A.N.); 2 Department of Biomedical Sciences, Colorado State University, Fort Collins, CO 80523, USA; whitcoml@ohsu.edu (L.A.W.); adam.chicco@colostate.edu (A.J.C.); 3 Department of Animal and Dairy Science, University of Georgia, Athens, GA 30602, USA; mgravelydavis@gmail.com (M.E.G.); hanna11ma@comcast.net (H.M.A.); alambarriod@uga.edu (D.A.A.); johngonz@uga.edu (J.M.G.); 4 Department of Animal Sciences, Colorado State University, Fort Collins, CO 80523, USA; colton.smith@colostate.edu (C.L.S.); mahesh.narayanan_nair@colostate.edu (M.N.N.); huey.loh@colostate.edu (H.Y.L.); terry.engle@colostate.edu (T.E.E.)

**Keywords:** nicotinamide riboside, porcine longissimus, mitochondrial function, apoptosis, postmortem muscle

## Abstract

Background/Objectives: Early postmortem mitochondrial function and apoptotic activation affect meat quality development. Nicotinamide riboside (NR) supplementation to pigs prior to harvest can improve pork color stability, but its mechanism remains unclear. This study aimed to evaluate the impact of NR supplementation on early postmortem mitochondrial functionality and apoptosis. Methods: Sixteen pigs (*N* = 16) were individually fed a control or NR-supplemented diet (30 mg·kg body weight^−1^·d^−1^) for 10 days prior to harvest. Longissimus dorsi muscle samples were collected at 45 min and 24 h postmortem and analyzed for mitochondrial functionality using high-resolution respirometry and apoptotic protein abundance (apoptosis regulator Bcl-2-associated X (BAX), apoptotic inducing factor (AIF), and caspase 3 (CASP3)) via immunoblotting. Results: NR-supplemented muscle exhibited lower proton leak-associated respiration at 45 min postmortem (*p* < 0.05), followed by a slower accumulation of mitochondrial outer membrane permeabilization (MOMP; *p* < 0.05) and a slower loss of mitochondrial integral function (*p* < 0.05) from 45 min to 24 h postmortem. NR supplementation decreased BAX abundance at 45 min postmortem but increased mature AIF abundance (62 kDa) at 24 h postmortem (*p* < 0.05). The abundance of CASP3 fragments (~29 kDa) decreased from 45 min to 24 h postmortem, independent of treatment (*p* < 0.05). Conclusions: NR supplementation demonstrated the potential to protect mitochondrial integral function and alleviate apoptotic activation in early postmortem porcine skeletal muscle, which might contribute to a higher meat color stability in NR-supplemented pork during retail display.

## 1. Introduction

Nicotinamide riboside (NR) is a pyridine-nucleoside, a form of vitamin B3 (pyridine-3-carboxylic acid), which could serve as a precursor to nicotinamide adenine dinucleotide (NAD^+^). Previous studies have demonstrated that NR supplementation can improve mitochondrial function by reducing the accumulation of reactive oxygen species (ROS) [1,2,3,4] and can prevent apoptotic activation [3,4,5,6]. Similarly, the beneficial effects of NR supplementation have been reported to improve muscle performance [7] and increase lean body mass [8]. A recent study also indicates that NR dietary supplementation can be useful in preventing fatigued pig syndrome after arriving at the abattoir [9].

Mitochondria are the main organelles responsible for energy metabolism in animals, and they can significantly affect animal health and production efficiency [10,11]. Although mitochondria could maintain their structural integrity and consume oxygen in postmortem muscle for up to 60 days [12], mitochondrial outer membrane permeabilization (MOMP) will increase in skeletal muscle when the postmortem period extends [13], which further triggers caspase-dependent and caspase-independent apoptosis [14]. Earlier or greater apoptosis promotes meat tenderization via proteolysis [15] but accelerates meat discoloration by diminishing metmyoglobin-reducing activity [16]. Therefore, the factors regulating postmortem muscle apoptosis tended to cause meat quality variation.

A previous pilot study reported that NR supplementation to pigs (30 mg·kg body weight^−1^·d^−1^ for 10 days) increased the meat redness and color stability in *Longissimus dorsi* (LD) muscle during the retail display as compared with the results for the non-supplemented controls [17]. Mitochondrial function in postmortem muscle is a key regulator of meat quality. However, the impact of NR supplementation on postmortem mitochondrial functionality and apoptotic activation is still unknown. Apoptosis regulator Bcl-2-associated X (BAX) is a pro-apoptotic protein inducing MOMP [18,19], while the proteolytic patterns of apoptotic inducing factor (AIF) and caspase 3 (CASP3) reflect caspase-independent [20] and caspase-dependent apoptosis [21], respectively. Therefore, this study aimed to evaluate the effects of NR supplementation on mitochondrial functionality and apoptosis through BAX, AIF, and CASP3 in LD muscle during the early postmortem period.

## 2. Materials and Methods

### 2.1. Animal Care and Use

The Institutional Animal Care and Use Committee at the University of Georgia (approval number A2020 03-004-R2) approved all animal procedures.

### 2.2. Animal Information

The finishing barrows (*N* = 16; 95 ± 5.1 kg body weight; CG36 × P26; Choice Genetics, West Des Moines, IA, USA) were approximately 6 months old prior to the experiment. The pigs were housed in individual pens (5 × 1.5 m) equipped with feeders and waterers to allow ad libitum access to feed and water. After a 21-day pen acclimation, a control diet (conventional swine finishing diet without supplemental nicotinamide riboside; *n* = 8) or an NR supplementation diet (control diet with NR [ChromaDex; Los Angeles, CA, USA]; 30 mg·kg^−1^ body weight^−1^·d^−1^; *n* = 8) was administered to the pen the final 10 days before harvest. The control diet contains 79.43% corn, 16.2% soybean meal, 2.0% fat, 0.35% salt, 0.58% dicalcium phosphate, 0.24% lysine, and 0.5% swine pre-mix (3.6% zinc, 3.5% iron, 1.0% magnesium, 3200 ppm copper, 500 ppm iodine, 60 ppm selenium, 1,000,000 IU/lb vitamin A, 150,000 IU/lb vitamin D3, 4000 IU/lb vitamin E, and 3 mg/lb vitamin B12). No extra niacin, tryptophan, or NAD^+^ producers were added to the control diet. On day 5, average daily feed intake and body weights were used to calculate the NR supplementation rate from day 5 to day 10. On day 11, the pigs were transported to a USDA-inspected commercial slaughter facility. The animals were stunned and bled, according to commercial practice, under USDA inspection. Porcine longissimus dorsi (LD) muscle biopsies (3 g) were collected from the left side of the carcasses between the 10th and 12th rib at 45 min and 24 h post-exsanguination to evaluate the mitochondrial function and apoptotic protein abundances under initial apoptotic activation and after rigor completeness, consistent with the methods used in the previous literature [22,23,24,25], and coordinated with the availability of the slaughter facility. Upon collection, half of the sample was immediately placed into ice-cold biopsy preservation medium (BIOPS; pH 7.1) containing 10 mM Ca-EGTA (0.1 µM free calcium), 20 mM imidazole, 20 mM taurine, 50 mM K-MES, 0.5 DTT, 6.56 mM MgCl_2_, 5.77 mM ATP, and 15 mM phosphocreatine, whereas the other half of the sample was snap-frozen in liquid nitrogen for protein analysis.

### 2.3. Muscle Sample Preparation

Muscle fibers from the LD were prepared for high-resolution respirometry experiments, as described previously [26,27]. Briefly, the muscle fibers were teased in ice-cold BIOPS solution containing 10 mM Ca-EGTA (0.1 μM free calcium), 20 mM imidazole, 20 mM taurine, 50 mM K-MES, 0.5 mM DTT, 6.56 mM MgCl_2_, 5.77 mM ATP, and 15 mM phosphocreatine (pH 7.1) before incubation with 50 μg/mL saponin on ice for 20 min, with gentle rocking to permeabilize cell membranes, while leaving mitochondrial membranes intact, allowing externally added compounds access to the mitochondria [28]. The permeabilized fiber bundles were then transferred to a mitochondrial respiration medium (MiR05) containing 0.5 mM EGTA, 3 mM MgCl_2_ hexahydrate, 60 mM lactobionic acid, 20 mM taurine, 10 mM KH_2_PO_4_, 20 mM HEPES, 110 mM sucrose, and 0.1% BSA (pH 7.1) and rinsed by rocking for 10 min on ice, followed by another identical 15-min rinse. The fiber bundles were then gently blotted dry for 10 to 15 s on Whatman filter paper and weighed immediately before adding approximately 8 mg to the 2-mL oxygraph chamber for the experiments.

### 2.4. Mitochondrial Respiration

Mitochondrial respiratory function was determined in permeabilized muscle fiber bundles collected at 45 min and 48 h postmortem using high-resolution respirometry (HRR) with an Oxygraph-2k high-resolution respirometer (Oroboros Instruments, Innsbruck, Austria). Standardized instrumental and chemical background calibrations were applied using Datlab software 5.1.1.9 (Oroboros Instruments), which includes corrections for the background diffusion of oxygen into the chamber, the oxygen solubility in MiR05, and the background consumption of oxygen (non-mitochondrial oxygen consumption) by the electrodes across a broad range (50–450 μmol/L) of chamber O_2_ concentrations. Oxygen flux was monitored in real-time by resolving changes in the negative time derivative of the chamber oxygen concentration signal, normalized to fiber bundle weight. A description of the respiration protocols and associated respiratory states generated by the sequential titration of each substrate is provided in Table 1.

In detail, muscle fiber samples were added to the oxygraphy chamber immediately before the addition of 2 mL of 100% O_2_ gas to the air vortex created by stirring the chamber media with the stopper partially inserted, allowing media O_2_ concentration to increase rapidly. The chambers were fully closed when the media O_2_ concentration reached ~400 μmol/L. After initial signal stabilization, the mitochondria were energized with saturating concentrations of the substrates listed in Table 1, which fully reconstitutes the forward flux of the citric acid cycle, oxidative phosphorylation (OXPHOS), and Complex IV. After the substrate addition of each respirometry flux state, at least 15 measurements recorded at 4 s intervals were collected after the oxygen flux signal became stable. All the data collected were normalized by the weight of the permeabilized fiber bundles. All respirometry data were collected at 37 °C in a hyperoxygenated environment (275–400 μmol/L) to avoid potential limitations in oxygen diffusion in the permeabilized fiber bundles, enabling consistent comparisons across samples [26].

### 2.5. Immunoblot Analysis for Apoptotic Proteins

Immunoblot analysis was conducted as described previously [27]. Briefly, protein was isolated from the muscle samples in mammalian lysis buffer (150 mM NaCl, 1 mM EDTA, 1 mM EGTA, 5 mM sodium pyrophosphate, 1 mM sodium orthovanadate, 20 mM sodium fluoride, 50 mL of Mammalian Protein Extraction Reagent [Thermo Scientific, 78501], and 500 µL of Protease Inhibitor Cocktail [Thermo Scientific, Rockford, IL, 78442, USA]). The concentration of the protein was determined using the Bradford Protein Assay Kit (Bio-Rad, #5000205) through a Shimazu UV-1800 spectrophotometer (Shimadzu Inc., Candy, OR). Protein (20 μg) was loaded and separated using 13.5% sodium dodecyl sulfate polyacrylamide gel electrophoresis gel and Mini-PROTEAN systems (Bio-Rad, Hercules, CA, USA). The separated protein was transferred to a methanol-activated PVDF membrane, washed by TBST (0.24% [*w:v*] Tris-base, 0.88% [*w:v*] NaCl, 0.1% [*v:v*] Tween-20, pH 7.6 adjusted by 6 N HCl) and blocked in 5% [*w:v*] non-fat dry milk in TBST. The membrane was incubated in primary antibodies (rabbit anti-AIF [bs-0037R]; rabbit anti-BAX [bs-0127R]; rabbit anti-caspase-3 [bs-0081R]; 1:500 dilution) overnight at 4 °C and in secondary antibody (donkey anti-rabbit [Invitrogen 31458]; 1:5000 dilution) for 1 h at room temperature. After incubation in SuperSignal West Dura Extended Duration Substrate (Thermo Scientific, 34075) for 30 s, the membranes were imaged using the iBright CL750 imaging system (Invitrogen, Carlsbad, CA, USA). Band density was normalized to AmidoBlack total protein (Sigma, St. Louis, MO, USA, A8181), as suggested by Ref. [29], and analyzed by ImageJ software (1.54g).

### 2.6. Statistical Analysis

A split-plot design was used to evaluate the effects of NR supplementation and postmortem time on each mitochondrial respiration state (Table 1), outer mitochondrial membrane (OMM) damage (Table 1), and the abundance of each protein band. Data analysis was performed by R (version 4.3.2), using the lme4 package as a mixed model, where diet (control or NR), postmortem time (45 min or 24 h), and their interactions were fixed effects, and the random effect in the model was each individual animal. The differences between least-square means (*p* < 0.05) were determined by Tukey’s multiple comparisons.

## 3. Results

### 3.1. Muscle Mitochondrial Respiration

**Figure 1:**
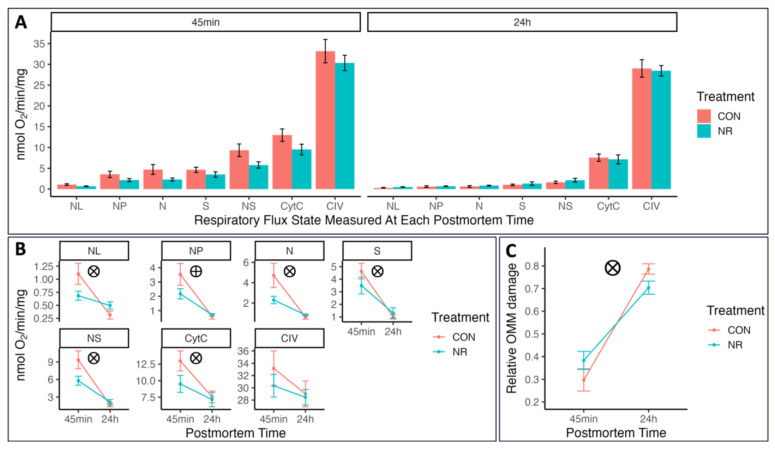
Muscle respiration under each respiratory flux state (**A**,**B**) and relative mitochondrial outer membrane damage (**C**) at 45 min and 24 h postmortem (*n* = 8). NL = proton leak-associated respiration supported by malate + pyruvate oxidation, but no ADP; NP = OXPHOS-linked-respiration supported by pyruvate + malate oxidation with ADP added; N = NP + glutamate oxidation; NS = N + succinate oxidation; S = NS − N; CytC = maximal mitochondrial integral respiration capacity supported by all substrates (NS) + cytochrome C; CIV = maximal oxygen reductase capacity of Complex IV using an artificial electron donor; Relative OMM damage = the lost integral respiration due to mitochondrial outer membrane permeabilization (calculated by [Cytc − NS]/[Cytc]). ⨂ = a significant PAP × postmortem-hour interaction (*p* < 0.05); ⨁ = a significant postmortem-hour effect (*p* < 0.05). Note: Detailed information for each respiratory flux state is presented in Table 1.

The mitochondrial respiratory capacity in permeabilized LD muscle fiber bundles from pigs supplemented with and without nicotinamide riboside (NR) under different substrate oxidation states is shown in Figure 1. Overall, CON muscle tended to exhibit higher mitochondrial respiratory rates than did NR muscle at 45 min postmortem (Figure 1) but rapidly declined to be similar to NR muscle by 24 h postmortem (*p* = 0.010, 0.029, 0.040, 0.013, and 0.017 for treatment × postmortem-time interaction in NL, N, S, NS, and CytC states). The differences were the most robust for the proton leak-associated respiration state (NL; under conditions of abundant substrate availability in the absence of ADP) at 45 min postmortem, which reflects a greater extent of proton leakage across the inner mitochondrial membrane in CON muscle [28,30].

Declines in OXPHOS-linked respiration states supported by pyruvate + malate (NP; *p* = 0.00004), succinate (S; *p* = 0.0000007), NADH + succinate (NS; *p* = 0.000001), and NADH + succinate + cytochrome c (CytC; *p* = 0.000007) from 45 min to 24 h postmortem were observed in both groups, indicating that maximal respiratory capacity declined after animal slaughter, independent of NR supplementation. Still, the decreases in the S, NS, and CytC respiratory states were greater in the CON group than in the NR group (*p* = 0.040, 0.013, and 0.017 for treatment × postmortem-time interaction). Similarly, although the relative outer mitochondrial membrane (OMM) damage increased from 45 min to 24 h postmortem, regardless of treatment, its accumulation was faster (*p* = 0.023) in the CON group than in the NR group. In addition, similar (*p* > 0.05) maximal complex IV enzymatic capacities (CIV) were seen between the CON and NR groups at 45 min and 24 h postmortem, suggesting that the proton leak-associated respiration and MOMP, and not the maximal complex IV capacity, accounted for the observed difference in N, S, NS, and CytC respirations between groups. Overall, the results indicated a greater proton leak-associated respiration and a faster collapse in mitochondrial function of the CON muscle than the NR muscle during the early postmortem period.

### 3.2. Immunoblot Analysis for Apoptosis Regulator Bcl-2-Associated X Protein, Apoptotic Inducing Factor, and Caspase 3 in LD Pig Muscle During the Early Postmortem Period

**Figure 2:**
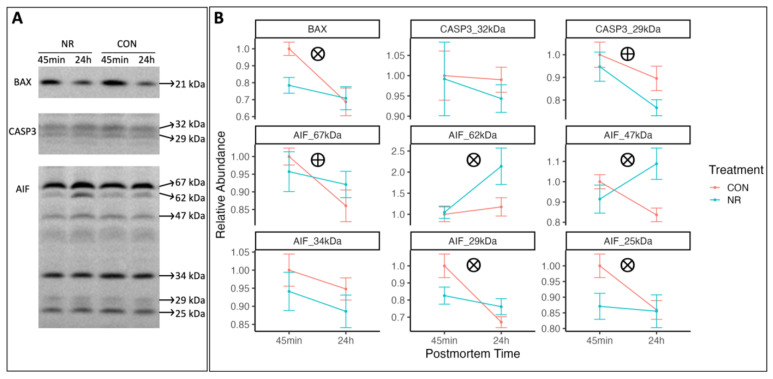
Representative Western blots (**A**) and the relative abundance (**B**) of apoptosis regulator Bcl-2-associated X (BAX), caspase 3 forms (CASP3 32 kDa and 29 kDa), and apoptotic inducing factor forms (AIF 67 kDa, 62 kDa, and ~47 kDa, ~34 kDa, ~29 kDa, and ~25 kDa) in pig longissimus dorsi muscle at 45 min and 48 h postmortem (n = 8). ⨂: a significant PAP × postmortem-hour interaction (*p* < 0.05); ⨁: a significant postmortem-hour effect (*p* < 0.05).

Targeting the ∆84-175 as the antigenic site of apoptosis regulator Bcl-2-associated X (BAX), a significant treatment × postmortem-time interaction was observed for the abundances of BAX (~21 kDa; *p* = 0.034; Figure 2). The CON muscle had a greater abundance of BAX than did the NR muscle at 45 min postmortem but rapidly declined to values similar to those of NR muscle by 24 h postmortem.

Utilizing an antibody targeting the ∆131-230 as the antigenic site of AIF, we identified six forms of AIF (67 kDa, 62 kDa, ~47 kDa, ~34 kDa, ~29 kDa, and ~25 kDa) during the early postmortem period (Figure 2). The 67 kDa form is the AIF precursor that can be imported into mitochondria, where it loses its mitochondrial leading sequence (MLS; ∆1–54) via proteolysis and becomes the 62 kDa mature form [20]. The mature form can generate the 57 kDa [20] and 47 kDa [31] fragments upon further proteolytic cleavage. AIF short 2 (AIFsh2) and AIF short 3 (AIFsh3) are two isoforms possessing partial sequences of AIF (∆1–322 and ∆87–322, respectively), and these were identified at molecular weights of ~34 kDa and ~25 kDa [32]. The MLS of AIFsh2 can be cleaved, yielding an AIFsh2 fragment without apoptotic activity (∆55–322; ~29 kDa) [32]. A main effect of postmortem-time (*p* = 0.036) was observed for the abundance of AIF precursor (67 kDa), with the abundance decreasing from 45 min to 24 h postmortem, independent of treatment. A significant treatment × postmortem-time interaction was observed for the abundance of the 62 kDa, ~47 kDa, ~29 kDa, and ~25 kDa forms of AIF (*p* = 0.041, 0.00024, 0.013, and 0.049; Figure 2). NR muscle displayed a similar abundance of the 62 kDa and ~47 kDa forms of AIF with that of CON muscle at 45 min postmortem but rapidly increased to be higher than CON muscle by 24 h postmortem (*p* = 0.041 and 0.00024). In comparison, CON muscle showed greater abundances of the ~29 kDa and ~25 kDa forms of AIF than did the NR group, but this rapidly declined to be similar to that of the NR group by 24 h postmortem (*p* = 0.013 and 0.049). Nearly identical abundances of the ~34 kDa form of AIF were seen between the CON and NR groups from 45 min and 24 h postmortem (*p* > 0.05).

Targeting the ∆1–100 of caspase 3, we identified two forms of caspase 3 (32 kDa and 29 kDa). Depending on the cell’s ATP status, the 32 kDa full-length caspase 3 can be further cleaved into 29 kDa or 17 kDa and 12 kDa [33]. Nearly identical abundances of full-length caspase 3 (32 kDa) were seen between the CON and NR groups from 45 min to 24 h postmortem (*p* > 0.05; Figure 2), while a main effect of postmortem-time (*p* = 0.0080) was observed for the abundance of the 29 kDa fragment of caspase 3, with the abundance decreasing from 45 min to 24 h postmortem.

## 4. Discussion

In this study, for the first time, to the best of our knowledge, we investigated the effects of NR supplementation on mitochondrial function and apoptotic biomarkers in the porcine LD muscle during the early postmortem period. Reactive oxygen species (ROS) are produced when mitochondrial electron leakage occurs during oxidative phosphorylation [34], and this production is maximized when proton leak-associated respiration (NL state) is high [26]. Lower proton leak-associated respiration and ROS accumulation resulting from NR treatment were recently observed in different research models [1,2,3,4,5,6]. These protective functions from NR were attributed to the activations of the Sirt1 [1,2,3], Sirt3 [4,6], and Jak2/Stat3 [5] pathways via the upregulation of NAD^+^ levels. Activation of these pathways can maintain mitochondrial inner potential [5] and keep proton leak-associated respiration low [1]. In agreement, muscle from the NR pigs exhibited lower proton leak-associated respiration (NL; Figure 1) than did the CON muscle at 45 min postmortem in the present study, indicating a lower ROS production rate in the NR muscle.

It is also worth noting that the difference in proton leak-associated respiration (NL; Figure 1) between the two treatment groups disappeared as postmortem time extended, and a much greater postmortem decline occurred in the CON muscle’s maximal OXPHOS-linked respiration states (N, S, NS, and CytC; Figure 1) than in those of the NR group. These reflect a faster loss of mitochondrial integral function (membrane integrity or/and protein function) in CON than NR muscle over time, which is likely due to the accumulated mitochondrial damage from ROS. Consistent with this interpretation, we observed a faster accumulation of OMM damage in the CON group than in the NR group from 45 min to 24 h postmortem (Figure 1), confirming the faster MOMP and thus, greater apoptosis activation in the CON group. Early postmortem skeletal muscle with a higher proton leak-associated respiration or greater abundance of ROS-producing enzymes leads to faster mitochondrial damage and earlier apoptosis during muscle-to-meat conversion and lower meat color stability during retail display [27,35]. Therefore, the alleviated mitochondrial ROS damage and lower apoptotic activation might partially explain the improved meat color stability in pork LD muscle due to NR supplementation [17].

Postmortem muscle cells commit cell death through apoptosis, and their apoptotic level increases when postmortem time extends [24,25]. To further confirm the effect of NR supplementation on apoptosis, we further evaluated the protein abundance of apoptosis regulator Bcl-2-associated X (BAX), apoptotic inducing factor (AIF), and caspase 3 (CASP3) via immunoblotting. BAX is a proapoptotic protein that can be activated by an increased ROS level [36] and decreased pH [37]. Under severe apoptotic stimulation, BAX can increase its abundance in the protein level [18,38] and directly cause the MOMP to engage in apoptosis [19]. Recent studies have indicated that NR treatment lowered BAX abundance and apoptosis rate via lower mitochondrial ROS production [4,6]. Thus, the higher abundance of BAX in CON than in NR muscle at 45 min postmortem indicated a greater apoptotic activation, and this upregulation is likely due to the higher ROS production rate (NL state; Figure 1) in the CON group. Considering the proapoptotic nature of BAX, the higher BAX abundance at 45 min might also have contributed to the faster accumulation of MOMP in the CON group from 45 min to 24 h postmortem (OMM damage; Figure 1).

The proteolysis of apoptotic inducing factor (AIF) reflects the AIF-related apoptosis, independent of caspase [20]. The current study evaluated the AIF proteolysis in postmortem porcine muscle and identified six AIF isoforms from 45 min to 24 h postmortem. Five forms (67 kDa, 62 kDa, ~47 kDa, ~29 kDa, and ~24 kDa) changed abundances from 45 min to 24 h postmortem, and the changes in four forms (62 kDa, ~47 kDa, ~29 kDa, and ~24 kDa) were treatment-dependent. Mature AIF (62 kDa) has the cochaperone function of maintaining the OXPHOS system function by protecting proper OXPHOS protein folding [39]. This is consistent with our results, where a slower loss of mitochondrial OXPHOS function (Figure 1) concurs with an increased abundance of mature AIF (62 kDa) in the NR group during the early postmortem period (Figure 2). Likewise, a previous study observed a protective unfolded protein response in mitochondria stimulated by NR supplementation [40]. However, the biological functions of the other AIF forms were not well understood at this time. These data suggested that caspase-independent apoptosis existed in porcine skeletal muscle during the early postmortem period and could be alleviated by NR supplementation. NR supplementation caused an increased abundance of mature AIF from 45 min to 24 h postmortem, which might have contributed to the slower loss of OXPHOS function under apoptotic activation. Still, the mechanism of NR supplementation’s effect on AIF proteolysis is unknown and warrants further investigation.

Upon cleavage, caspase 3 can function as cysteine–aspartic acid protease that cleaves cellular targets and executes caspase-dependent apoptotic cell death [21]. Independent of NR treatment, the change in the caspase 3 fragment (~29 kDa) was observed in porcine muscle from 45 min to 24 h postmortem, which echoes that caspase 3 was involved in postmortem apoptosis [15]. However, as in other postmortem muscle studies [15,22], our experiment did not identify the canonical cleavage fragment (~17 kDa). This atypical pattern of caspase-3 cleavage is possibly due to ROS-induced cellular ATP depletion [33]. This also corresponds with the rapid decline of mitochondrial integral function at postmortem in both treatment groups (NP, N, S, NS, and CytC states; Figure 1).

## 5. Conclusions

NR supplementation demonstrated potential for affecting mitochondrial integral function and apoptotic activation in early postmortem porcine skeletal muscle. NR muscle showed a slower early postmortem accumulation of mitochondrial outer membrane permeabilization and a slower loss of mitochondrial integral function. The alleviated early postmortem mitochondrial damage rate in NR muscle could be attributed to the lower mitochondrial ROS production, the lower BAX-dependent mitochondrial outer membrane permeabilization, and the higher AIF cochaperone function. The protected mitochondrial integrity might contribute to a higher meat color stability in NR-supplemented pork during retail display. Still, future larger-scale studies are warranted to comprehensively evaluate how NR supplementation affects mitochondrial function, apoptosis, and meat quality development during broader postmortem periods in various porcine breeds.

## Figures and Tables

**Table 1:** High-resolution respirometry protocols and associated respiratory flux states assessed in the mitochondrial respiration experiment.

Protocol Titrations (Final Concentration in Chamber)	Abbreviation and Explanation of Each High-Resolution Respirometry Flux State
Malate (1 mM) + Pyruvate (5 mM)	NL: Proton leak-associated respiration supported by high NADH (pyruvate + malate oxidation), but no ADP.
ADP (2.5 mM)	NP: OXPHOS-linked respiration supported by NADH (pyruvate + malate oxidation) in the presence of ADP.
Glutamate (10 mM)	N: Maximal OXPHOS-linked respiration supported by NADH (malate + pyruvate + glutamate oxidation) with ADP.
Succinate (10 mM)	NS: Maximal OXPHOS-linked respiration supported by NADH and succinate with ADP.
	S: Maximal OXPHOS-linked respiration supported by succinate after subtracting previous N-state rate (calculated by NS − N).
Cytochrome C (4 mM)	CytC: Maximal mitochondrial integral respiration capacity supported by NADH + succinate + cytochrome C.
	Relative OMM damage: The lost integral respiration due to mitochondrial outer membrane permeabilization (MOMP; calculated by [CytC − NS)]/CytC).
Ascorbate (2 mM) + TMPD (0.5 mM)	CIV: Maximal oxygen reductase capacity of Complex IV using an artificial electron donor.

## Data Availability

The datasets generated and/or analyzed during the current study are not publicly available but are available from the corresponding author upon reasonable request.
